# The right to health-care and the regionalization of the health-care system

**DOI:** 10.1186/1824-7288-40-S1-A88

**Published:** 2014-08-11

**Authors:** Stefano Semplici

**Affiliations:** 1 Department of Business Government Philosophy Studies, University of Rome “Tor Vergata”, Rome, 00173, Italy

## 

In Italy, the process of decentralization of the health-care organization was given a strong boost during the last decade of the twentieth century, according to some inspiring lines of the Law itself, which established in 1978 the National Healthcare Service. Regions were given more and more autonomy and legislative powers in this field, so that they gained a substantial role in programming, organizing and managing health care services. The new Article 117 of the Constitution, adopted in 2001, made it explicit that health protection is a matter of concurring legislation, with the clarification that in such matters the State, which maintains exclusive powers with regard to the determination of the basic level of benefits relating to civil and social entitlements, is given the role to lay down only the fundamental principles. As a consequence, the role of the State – as it is affirmed on the website of the Italian Ministry of Health – has changed into a function of warrantor of fairness, building on the concept of the essential levels of assistance which ought to be guaranteed throughout the national territory.

The major risks that deserve a special attention are those concerning the possibility of a too deep fault of inequality as to what is offered in different regions beyond the threshold of what is established as essential at the national level and a too broad discretion in defining standards, procedures, guidelines, not to mention the burning issues of costs and quality. Paediatrics, far from being an exception, offers telling examples of the asymmetries and gaps that we should fill in, in order not to miss the obligation entailed in the recognition of the right to health-care as a constitutional essential. The neonatal screening programmes on the one side and the management of vaccination on the other side underline the difficulty of drawing the line of what is essential as well as acknowledging the limits of regional autonomy in applying fundamental principles. The situation of neonatal intensive care units underlines faults of inequality that look hard to reconcile with the idea of one and the same dignity of all citizens. Needless to say, starting with the newborn.

